# A gain‐of‐function *GRIA2* variant associated with neurodevelopmental delay and seizures: Functional characterization and targeted treatment

**DOI:** 10.1111/epi.17419

**Published:** 2022-10-09

**Authors:** Ian D. Coombs, Julie Ziobro, Volodymyr Krotov, Taryn‐Leigh Surtees, Stuart G. Cull‐Candy, Mark Farrant

**Affiliations:** ^1^ Department of Neuroscience, Physiology, and Pharmacology University College London London UK; ^2^ Department of Pediatrics University of Michigan Ann Arbor Michigan USA; ^3^ Department of Neurology Washington University in St Louis School of Medicine St Louis Missouri USA

**Keywords:** AMPA receptor, epilepsy, GluA2, *GRIA* disorder, perampanel

## Abstract

α‐Amino‐3‐hydroxy‐5‐methyl‐4‐isoxazolepropionic acid‐type glutamate receptors (AMPARs) are ligand‐gated cationic channels formed from combinations of GluA1‐4 subunits. Pathogenic variants of *GRIA1–4* have been described in patients with developmental delay, intellectual disability, autism spectrum disorder, and seizures, with *GRIA2* variants typically causing AMPAR loss of function. Here, we identify a novel, heterozygous de novo pathogenic missense mutation in *GRIA2* (c.1928 C>T, p.A643V, NM_001083619.1) in a 1‐year‐old boy with epilepsy, developmental delay, and failure to thrive. We made patch‐clamp recordings to compare the functional and pharmacological properties of variant and wild‐type receptors expressed in HEK293 cells, with and without the transmembrane AMPAR regulatory protein γ2. This showed GluA2 A643V‐containing AMPARs to exhibit a novel gain of function, with greatly slowed deactivation, markedly reduced desensitization, and increased glutamate sensitivity. Perampanel, an antiseizure AMPAR negative allosteric modulator, was able to fully block GluA2 A643V/γ2 currents, suggesting potential therapeutic efficacy. The subsequent introduction of perampanel to the patient's treatment regimen was associated with a marked reduction in seizure burden, a resolution of failure to thrive, and clear developmental gains. Our study reveals that *GRIA2* disorder can be caused by a gain‐of‐function variant, and both predicts and suggests the therapeutic efficacy of perampanel. Perampanel may prove beneficial for patients with other gain‐of‐function *GRIA* variants.

## 1 INTRODUCTION

α‐Amino‐3‐hydroxy‐5‐methyl‐4‐isoxazolepropionic acid‐type glutamate receptors (AMPARs)—homo‐ or heterotetrameric assemblies of the subunits GluA1–4, encoded by *GRIA1–4*—are ligand‐gated cation‐permeable ion channels that mediate excitatory synaptic transmission throughout the brain.^1^ They are critical for the correct development of neuronal circuitry, and changes in their number or function underlie activity‐dependent strengthening or weakening of synaptic signaling.^2^, ^3^ The most prevalent AMPAR subtypes are calcium‐impermeable di‐ or triheteromers containing GluA2.^4^, ^5^

To date, 27 de novo *GRIA2* variants have been identified in 31 patients with epilepsy, intellectual disability, neurodevelopmental disorders, and autism spectrum disorder.^6^, ^7^, ^8^ Electrophysiological investigation of these variants shows primarily AMPAR loss of function.^6^ Here, we describe a patient with a novel *GRIA2* variant within the SYTANLAAF motif that forms the channel activation gate (c.1928 C>T, p.A643V, NM_001083619.1),^1^, ^9^ who presented with medically refractory seizures, developmental delay, and failure to thrive. To guide treatment, we used electrophysiology to assess the functional effects of GluA2 A643V. This revealed gain of function and effective inhibition by perampanel, a selective AMPAR negative allosteric modulator (NAM) and antiseizure medication.^10^ We present real‐life treatment data on the clinical effects of perampanel in this patient.

## 2 MATERIALS AND METHODS

The study was deemed "not regulated" by the institutional review board (IRB) of the University of Michigan (UoM); publishing clinical findings from a single individual does not fit the definition of human‐subjects research requiring IRB approval (per 45 CFR 46, 21 CFR 56, and UoM policy). Written informed parental consent for genetic testing and publication was obtained. Trio genetic analysis (EpiXpanded Panel) was performed by GeneDX (https://www.genedx.com/tests/detail/epixpanded‐panel‐835). Developmental progress was ascertained by chart review of clinical notes (produced by the patient's neurologist and therapists) and family reports (age = 7–33 months). Seizure burden (from ages 20 to 33 months) was evaluated from daily seizure diaries kept by the patient's family. Concurrent antiseizure medication doses were kept stable during perampanel initiation. Levetiracetam was weaned after 2 months, following improved seizure control. Electroencephalographic (EEG) evaluations were performed as clinically indicated. Recording of glutamate‐evoked currents from heterologously expressed AMPARs in outside‐out membrane patches was performed as previously described (for full details and analysis methods see Methods S1).^11^

## 3 RESULTS

### 3.1 Clinical findings

**FIGURE 1:**
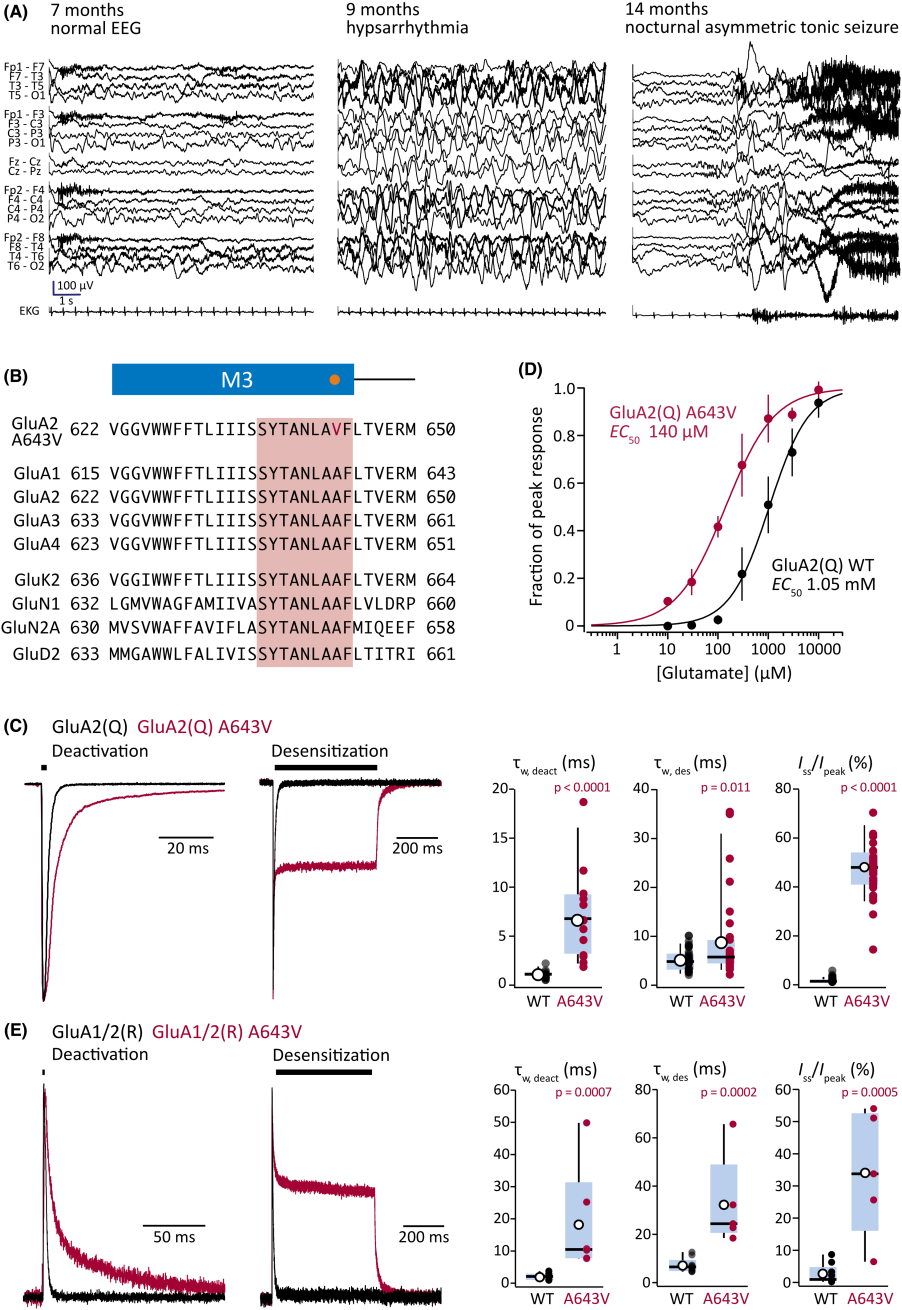
Clinical presentation and α‐amino‐3‐hydroxy‐5‐methyl‐4‐isoxazolepropionic acid‐type glutamate receptor (AMPAR) gain of function with the *GRIA2* A643V variant. (A) Electroencephalographic (EEG) findings. Progression is seen from normal EEG at 7 months of age (left) to hypsarrhythmia (middle) with infantile spasms (right) at 9 months of age. Hypsarrhythmia resolved with adrenocorticotropic hormone therapy, but frequent nocturnal asymmetric tonic seizures (right) developed at 14 months of age. (B) Sequence alignments highlighting the position of Ala643 (orange dot) in the third membrane region (M3; blue bar) of the AMPAR subunit. The surrounding region is completely conserved between all four AMPAR subunits, and the SYTANLAAF motif (pink box) is conserved throughout the iGluR superfamily. The gene sequences are from human GluA1 (NP_001107655.1), human GluA2 (NP_000817.5), human GluA3 (NP_015564.5), human GluA4 (NP_000820.4), human GluK2 (NP_068775.1), human GluN1 (NP_015566.1), human GluN2A (NP_000824.1), and human GluD2 (NP_001501.2). (C) Representative deactivating and desensitizing outside‐out patch responses (10 mmol·L^–1^ glutamate, 1 and 500 ms, −60 mV; black bars) from HEK293 cells transfected with wild‐type (WT) GluA2(Q) (black) or GluA2(Q) A643V (red; superimposed). Right: pooled weighted time constant of deactivation (τ_w, deact_) data for GluA2(Q) (*n* = 16) and GluA2(Q) A643V (*n* = 13) from 1‐ms glutamate applications, together with pooled desensitization time constant (τ_w, des_) and residual current (steady‐state current at end of application divided by peak current; *I*_ss_/*I*_peak_) data for GluA2(Q) (*n* = 31) and GluA2(Q) A643V (*n* = 33) from 500‐ms glutamate applications. Box‐and‐whisker plots indicate the median (black line), the 25–75th percentiles (pale blue box), and the 10–90th percentiles (whiskers); filled circles are data from individual patches, and open circles indicate means. (D) Pooled normalized concentration–response curves for GluA2(Q) (*n* = 5) and GluA2(Q) A643V (*n* = 5). Symbols and error bars indicate mean values with SD, and the solid lines are fits to the Hill equation, yielding the indicated half‐maximal effective concentration (EC_50_) values. Table 1 reports the statistical analysis of EC_50_ values obtained from separate fits of the data from individual patches. (E) Representative deactivating and desensitizing responses (10 mmol·L^–1^ glutamate, 1 and 500 ms, +60 mV; black bars) from heteromeric receptors in cells transfected with GluA1 and GluA2(R) (black) or GluA1 and GluA2(R) A643V (red; superimposed). Right: pooled τ_w, deact_ data for WT GluA1/GluA2(R) (*n* = 7) and GluA1/GluA2(R) A643V (*n* = 6), together with pooled τ_w, des_ and *I*_ss_/*I*_peak_ data for GluA1/GluA2(R) (*n* = 9) and GluA1/GluA2(R) A643V (*n* = 5). Boxplots as in C. Indicated *p*‐values are from two‐sided approximate permutation *t*‐tests comparing WT and A643V variant (Table 1).

Our patient was born via cesarian section at 37 weeks' gestation, following a pregnancy complicated by polyhydramnios. Apgar scores were 8 and 9 at 1 and 5 min. Following delivery, the patient required oxygen by nasal cannula for 3 h due to acute hypercapnic and hypoxic respiratory failure, which subsequently resolved. He was discharged at 3 days of age. At 2 months, he was diagnosed with gastroesophageal reflux disease (confirmed by swallow study), but maintained growth along the 50th percentile while treated with ranitidine and omeprazole. At 6 months, he was evaluated by an ophthalmologist for intermittent exotropia and poor visual tracking, and at 7 months by a pediatric neurologist for episodes of back arching and vomiting after feeds. His examination at that time showed decreased muscle tone and mild developmental delay. He was able to tripod sit, but unable to sit unsupported. He babbled appropriately, but was not clearly responsive to his name. A routine 30‐min EEG was normal (Figure 1A).

At 9 months, the patient presented to the emergency department with a fever, decreased interaction, and dystonic posturing with back arching and upward eye deviation followed by generalized body limpness. EEG monitoring showed hypsarrhythmia and captured multiple clusters of epileptic spasms (Figure 1A), both of which were resolved with adrenocorticotropic hormone and levetiracetam. Genetic analysis revealed a heterozygous de novo variant in *GRIA2* (c.1928 C>T, p.A643V, NM_001083619.1) located at the *lurcher* site^9^ in the conserved SYTANLAAF sequence (Figure 1B). The patient was admitted at 11 months for status epilepticus with focal impaired awareness seizures and intermittent focal tonic seizures arising from the right temporal region in the setting of respiratory syncytial virus infection, requiring introduction of phenobarbital. His development plateaued during this period. At 13 months, he was still unable to sit unsupported, had limited visual tracking and eye contact, and no specific words. Focal tonic seizures from sleep developed at 14 months (Figure 1A). Meanwhile, he developed significant vomiting and constipation, resulting in failure to thrive; his weight fell from the 63rd percentile (6 months) to the .07th percentile (20 months). Seizures were not improved by brief trials of valproic acid or clobazam, and a modified Atkins diet (MAD) was introduced.

### 3.2 GluA2 A643V causes gain of function

To determine the functional impact of the A643V variant, we expressed GluA2(Q) wild‐type or A643V subunits in HEK293 cells and compared outside‐out patch responses to glutamate (Figure 1C). For currents evoked by short glutamate applications (10 mmol·L^–1^, –60 mV, 1–2 ms), the weighted time constant of deactivation (τ_w, deact_) was approximately sevenfold greater with A643V compared to wild type (Figure 1C; Table 1). With long glutamate applications (500 ms), the desensitization time constant (τ_w, des_) was almost double that of wild type and the residual current (steady‐state current at end of application divided by peak current; *I*_ss_/*I*_peak_) was increased ~50‐fold (Figure 1C, Table 1); thus, desensitization was greatly reduced. Concomitantly, recovery from desensitization was faster for A643V than for wild type (Table 1). Furthermore, we found increased glutamate potency at A643V AMPARs (with the half‐maximal effective concentration reduced by approximately eightfold; Figure 1D), whereas channel conductance and open probability were unaffected (Table 1).

**TABLE 1:** Summary of GluA2 A643V effects on homomeric and heteromeric AMPARs expressed with and without transmembrane AMPAR regulatory protein γ2

	GluA2(Q)	GluA2(Q) A643V	Unpaired mean difference [95% CI]	*p*
τ_w, deact_, ms	1.0 ± .4 (16)	6.9 ± 4.6 (13)	5.9 [4.0, 9.0]	<.0001
τ_w, des_, ms	5.1 ± 2.2 (31)	9.5 ± 9.5 (33)	4.4 [1.7, 8.3]	.011
*I*_ss_/*I*_peak_, %	.9 ± 1.0 (31)	47.4 ± 11.7 (33)	46.5 [42.2, 50.3]	<.0001
Conductance, pS	10.5 ± 1.8 (7)	9.5 ± 1.7 (8)	−1.0 [−2.6, .7]	.29
*P*_o, peak_	.74 ± .12 (7)	.63 ± .11 (8)	−.11 [−.21, .01]	.11
Glu EC_50 peak_, mmol·L^–1^	1.14 ± .45 (5)	.15 ± .04 (5)	−.99 [−1.29, −.59]	<.0001
	GluA1/2(R)	GluA1/2(R) A643V		
τ_w, deact_, ms	2.0 ± 1.1 (7)	18.6 ± 16.7 (6)	16.6 [7.8, 36.8]	.0007
τ_w, des_, ms	7.2 ± 3.0 (9)	32.7 ± 19.1 (5)	25.5 [15.3, 50.4]	.0002
*I*_ss_/*I*_peak_, %	2.6 ± 3.0 (9)	34.2 ± 19.5 (5)	31.6 [15.1, 45.6]	.0005
	GluA2(Q)/γ2	GluA2(Q) A643V/γ2		
τ_w, deact_, ms	3.6 ± 2.0 (11)	50.5 ± 29.8 (17)	46.9 [35.9, 64.1]	<.0001
τ_w, des_, ms	13.6 ± 4.1 (29)	11.6 ± 13.7 (36)	−2.0 [−5.6, 4.2]	.48
*I*_ss_/*I*_peak_, %	14.1 ± 11.6 (29)	66.2 ± 10.2 (37)	52.1 [46.2, 57.2]	<.0001
PER IC_50 peak_, μmol·L^–1^	.13 ± .05 (6)	1.29 ± .77 (4)	1.17 [.66, 1.98]	.0052
PER IC_50 ss_, μmol·L^–1^		3.08 ± .51 (4)		
	GluA1/2(R)/γ2	GluA1/2(R) A643V/γ2		
τ_w, deact_, ms	10.0 ± 8.7 (9)	28.0 ± 13.6 (8)	18.0 [7.71, 28.2]	.0059
τ_w, des_, ms	7.8 ± 2.1 (9)	12.6 ± 9.9 (5)	4.8 [‐1.53, 14.7]	.16
*I*_ss_/*I*_peak_, %	10.5 ± 7.4 (9)	25.0 ± 9.1 (5)	14.4 [6.9, 23.9]	.0085

*Note*: Data are presented as mean ± SD with the number of replicates in parentheses. For GluA2(Q) A643V/γ2, the PER IC_50_ is shown for both the initial peak and ss currents. Unpaired mean differences are given with their 95% bias corrected and accelerated CIs [upper bound, lower bound], calculated from 5000 bootstrap resamples. All *p*‐values were calculated using a nonparametric two‐sided approximate permutation *t*‐test, with 10 000 bootstrap replicates. The *p*‐values are reported as equalities, unless <.0001 (see Methods S1). 1

We next examined the impact of GluA2 A643V on the prevalent heteromeric receptor GluA1/GluA2(R).^5^ As with homomeric receptors, we found that the variant slowed deactivation and markedly reduced desensitization (Figure 1E). Thus, τ_w, deact_ was increased nearly 10‐fold, τ_w, des_ more than fourfold, and *I*_ss_/*I*_peak_ more than 10‐fold (Table 1). In the presence of a key AMPAR auxiliary protein—transmembrane AMPAR regulatory protein γ2^1^—A643V also produced gain of function. Thus, with both homomeric and heteromeric γ2‐associated AMPARs, deactivation was similarly slowed and steady‐state desensitization was similarly decreased (Table 1).

### 3.3 In vitro and clinical effects of perampanel

**FIGURE 2:**
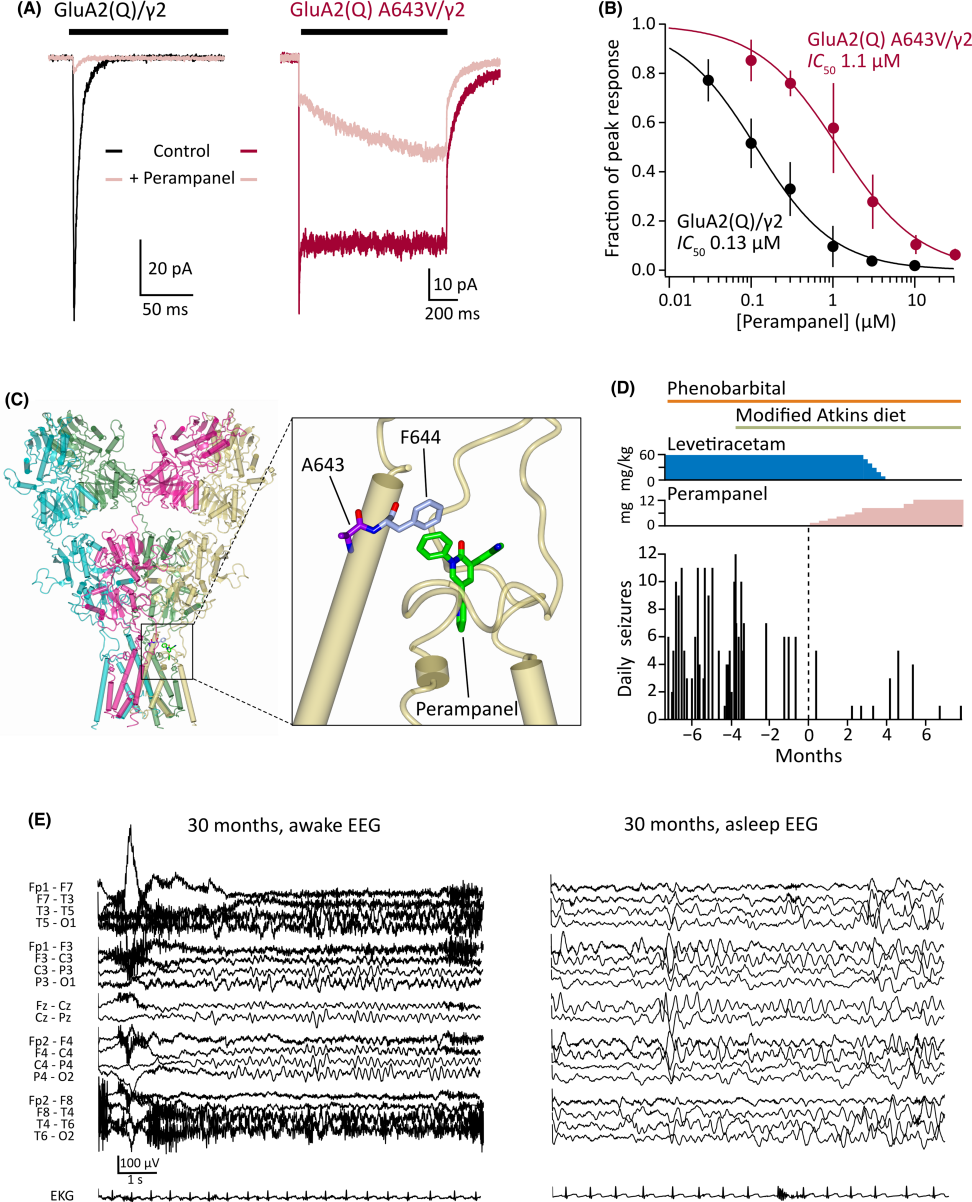
Functional and clinical impact of perampanel. (A) Representative GluA2(Q)/γ2 (black) and GluA2(Q) A643V/γ2 currents (red) with inhibition by 3 μmol·L^–1^ perampanel (pink). (B) Concentration inhibition curves demonstrating decreased potency of perampanel against the A643V variant (*n* = 6 for GluA2[Q]/γ2 and 4 for GluA2[Q] A643V/γ2). Symbols and error bars indicate mean values with SD, and the solid lines are fits to the Hill equation, yielding the indicated half‐maximal inhibitory concentration (IC_50_) values. Table 1 reports the statistical analysis of IC_50_ values obtained from separate fits of the data from individual patches. (C) Crystal structure of a GluA2(Q) receptor with perampanel bound, showing the binding site (including F644, which interacts with the phenyl ring of perampanel) adjacent to A643 (Protein Data Bank: 5L1F^12^). (D) Number of parent‐reported daily seizures in the months before and after commencement of perampanel treatment (at 0 months). Colored bars and plots denote the timing of antiseizure medications. Phenobarbital (6 mg/kg daily) is currently being weaned, whereas the modified Atkins diet is being maintained. Following the introduction of perampanel, levetiracetam was gradually withdrawn. (E) Electroencephalograms (EEGs) at 30 months of age following treatment with perampanel for 3 months. The awake (left) and sleep (right) interictal patterns remain slow and disorganized, with multifocal spikes in sleep, consistent with an epileptic encephalopathy with ongoing risk for seizures.

As the A643V variant caused gain of function, we tested the AMPAR NAM perampanel^10^ and found that it fully inhibited both peak and steady‐state glutamate responses of GluA2(Q) A643V/γ2 (Figure 2A,B, Table 1). This occurred with reduced potency compared to wild‐type GluA2(Q)/γ2, likely due to the proximity of the perampanel binding site to position 643 (Figure 2C).^12^ Nonetheless, this finding confirmed the potential for rescue pharmacology with perampanel.

Adjunctive treatment with perampanel was initiated at 27 months of age (1 mg/day, with gradual uptitration of the dose). Prior to this, at 24 months, introduction of MAD resulted in an initial drop in seizure number and frequency, although occasional (1–3/month) clusters of 6+ seizures persisted. Perampanel introduction was associated with a further reduction in seizure frequency (Figure 2D). In the 7 months prior to perampanel initiation, seizures occurred an average of 4.9 days per month (range = 1–10) and generally clustered with up to 12 nightly seizures. In the 7 months following perampanel administration, the patient had an average of only 1.1 seizure‐days per month (range = 0–2), with seizure clusters provoked by illness or teething. Given concomitant treatment with phenobarbital (a strong CYP3A4 inducer), perampanel clearance was expected to be increased, and thus we prescribed higher dosing than typical at this age. The patient was closely monitored for side effects including somnolence, irritability, ataxia, and elevated hepatic enzymes during perampanel titration, and safely reached the maximum dose of 12 mg/day. With improved seizure control, the patient was weaned off levetiracetam and phenobarbital has been reduced (Figure 2D). At 32 months, MAD was supplemented with medium‐chain triglyceride (MCT) oil.^13^

Perampanel introduction coincided with an acceleration in developmental gains; the patient gained the ability to sit independently, weight‐bear in quadrupedal position, and displayed improved fine motor, visual, and communication skills. Constipation, vomiting, and failure to thrive resolved, with weight (at 34 months) recovering to the 15.8th percentile. His family reports a significant improvement in baseline irritability, and, within the first 2 h of his perampanel dose, he displays consistent smiling and purposeful laughing, behaviors not previously observed. Nonetheless, the EEG at 3 months postperampanel remained slow and disorganized, with multifocal spikes predominantly over the bilateral posterior and left temporal regions, suggestive of an epileptic encephalopathy with ongoing risk for recurrent seizures (Figure 2E).

## 4 DISCUSSION

### 4.1 *GRIA* disorder

*GRIA* disorder is an emerging neurological disease with 100+ variants identified across *GRIA1–4*.^1^ Our patient exhibited several features previously reported for others with *GRIA2* disorder, including the onset of neurological symptoms at ~6 months, epilepsy, and developmental delay.^6^, ^7^, ^8^ However, certain symptoms were novel, including gastrointestinal disturbance and associated failure to thrive. Our study establishes *GRIA2* A643V as a gain‐of‐function variant, a previously unreported molecular phenotype that could be expected to account for the neurological symptoms observed.

The A643V variant modifies the SYTANLAAF motif, the most highly conserved sequence in the ionotropic glutamate receptor superfamily. This forms the upper channel gate with all four Ala643 residues of the tetramer in close proximity.^14^ As these Ala643 residues are tightly packed, the introduction of a larger sidechain (valine) is expected to destabilize the closed gate. It follows that channel closure by deactivation is likely to be less favorable and slower, and the degree of desensitization would be reduced.

### 4.2 Perampanel

We found perampanel to be much less potent on GluA2 A643V than on wild‐type receptors, likely due to the Val643‐induced rearrangements displacing the adjacent Phe644, which is indispensable for perampanel binding.^12^ Inhibitors that bind to alternative regions of the AMPAR might display improved selectivity against GluA2 A643V. Interestingly, decanoic acid, one component of the MCT ketogenic diet, may inhibit AMPARs by acting at a site distinct from that of perampanel.^15^ When used together, perampanel and decanoic acid act synergistically to provide enhanced suppression of seizurelike activity recorded in vitro from rodent and human brain tissue.^16^ Our patient's current treatment regimen—perampanel together with an MCT‐supplemented MAD—would therefore be expected to inhibit AMPARs through two different sites, which could explain its success despite perampanel's reduced relative potency.

Commonly reported perampanel side effects include dizziness and sedation, as well as negative mood alteration and aggression.^17^, ^18^ Remarkably, our patient displayed quite the opposite behavior, showing increased alertness with improving fine motor control, and reported mood improvement. A parsimonious explanation is that, when typically prescribed, perampanel produces global AMPAR underactivity, whereas in a patient with AMPAR gain of function, it may instead promote a normalization of AMPAR‐mediated excitation. It is also possible that the effects of perampanel will be influenced by the diversity of auxiliary subunits associated with different AMPAR populations, as is apparent for AMPAR positive allosteric modulators.^19^ Notably, the gastrointestinal symptoms and failure to thrive resolved. Whether this reflects perampanel's inhibition of overactive variant‐containing receptors (centrally or in the peripheral/enteric nervous system) is unclear. The decreased seizure burden after addition of perampanel allowed weaning from levetiracetam (complete) and phenobarbital (ongoing), which may have contributed to the positive outcome.

It is important to note that clinical management decisions for our patient were not made as part of a placebo‐controlled, double‐blinded study. Perampanel was added once in vitro results confirmed gain of function and probable safety. The lack of a placebo control and the use of parent reporting may lead to significant ascertainment bias in noting the overall effect of perampanel. Future follow‐up will provide continued insight as to the benefit of perampanel for patients with *GRIA2* gain‐of‐function variants, and potentially those with other *GRIA* gain‐of‐function variants.

## AUTHOR CONTRIBUTIONS

Conceptualization: Ian D. Coombs, Stuart G. Cull‐Candy, Mark Farrant. Formal analysis: Ian D. Coombs, Volodymyr Krotov, Mark Farrant. Funding acquisition: Stuart G. Cull‐Candy, Mark Farrant. Investigation: Ian D. Coombs, Volodymyr Krotov, Julie Ziobro. Project administration: Ian D. Coombs, Julie Ziobro, Mark Farrant. Writing–original draft: Ian D. Coombs, Stuart G. Cull‐Candy, Mark Farrant. Writing–review & editing: Julie Ziobro, Taryn‐Leigh Surtees, Ian D. Coombs, Stuart G. Cull‐Candy, Mark Farrant.

## CONFLICT OF INTEREST

None of the authors has any conflict of interest to disclose.

## PATIENT CONSENT

Written informed consent for genetic testing and publication was obtained from the patient's parents.

## Supporting information


APPENDIX S1

## Footnotes

1 Abbreviations: τ_w, deact_, weighted time constant of deactivation; τ_w, des_, desensitization time constant; *I*_ss_/*I*_peak_, steady‐state current at end of application divided by peak current; *P*_o, peak_, peak open probability; Gl, glutamate; PER, perampanel.
