# Supplementary material for: A gain‐of‐function GRIA2 variant associated with neurodevelopmental delay and seizures: Functional characterization and targeted treatment
Source: Epilepsia. 2022 Oct 9;63(12):e156–63. doi: 10.1111/epi.17419 (PMC10092096; doi:10.1111/epi.17419)
Supplement: Supplementary file 1 — APPENDIX S1 [file EPI-63-e156-s001.docx]

**SUPPLEMENTARY METHODS**

**Heterologous expression.** We expressed recombinant human AMPAR subunits and TARP γ2 (kind gifts from Dimitri Kullmann, UCL Queen Square Institute of Neurology, London, and Michael Maher, Janssen Research & Development L.L.C., San Diego, respectively), and EGFP, in HEK293 cells. These were maintained under standard protocols, as described previously ^1^. GluA1 and GluA2 subunit cDNAs were of the flip splice form. GluA2 subunit DNAs were R/G unedited and of the Q/R unedited form – GluA2(Q) – when expressed alone to give homomeric AMPARs or of the Q/R edited form – GluA2(R) – when expressed with GluA1 to give heteromeric AMPARs. Homomeric and heteromeric AMPARs were expressed both with- and without TARP γ2. The GluA2 A643V point mutation was produced using standard PCR protocols. AMPAR/TARP combinations were transfected at a cDNA ratio of 1:1. For GluA1/2 receptors, the subunits were expressed at a ratio of 1:2. Transient transfection was performed using Lipofectamine 2000 (Life Technologies), and electrophysiological recordings were performed 18-48 h later.

**Electrophysiology.** Recordings were made with an external solution containing 145 mM NaCl, 2.5 mM KCl, 1 mM CaCl_2_, 1 mM MgCl_2_, and 10 mM HEPES, pH 7.3. Patch-clamp electrodes were pulled from borosilicate glass (1.5 mm o.d., 0.86 mm i.d.; Harvard Apparatus) and fire polished to a final resistance of 8-12 MΩ. The internal solution contained 145 mM CsCl, 2.5 mM NaCl, 1 mM Cs-EGTA, 4 mM MgATP, and 10 mM HEPES (pH 7.3 with CsOH) and was supplemented with 100 μM spermine tetrahydrochloride or 100 μM NASPM for recordings from GluA2(Q) or GluA1/2, respectively. Recordings were made from outside-out patches at 22-25 °C using an Axopatch 200B amplifier (Molecular Devices). Currents were recorded at −60 mV for GluA2(Q) or +60 mV for GluA1/2, low-pass filtered at 10 kHz, and digitized at 20 kHz using an NI USB-6341 (National Instruments) interface with Strathclyde Electrophysiology Software WINWCP (John Dempster, University of Strathclyde, Glasgow UK).

**Rapid agonist application to excised patches.** Rapid agonist application was achieved by switching between continuously flowing solutions. Solution exchange was achieved by moving an application tool made from theta glass (Hilgenberg) or triple barreled glass (Vitrocom) mounted on a piezoelectric translator (Physik Instrumente). The 10-90% exchange times, assessed by jumping open electrodes into a diluted solution and observing junction potential changes, were between 150 and 300 μs.

Experiments to assess dose response relationships for glutamate were performed using triple barreled glass. The central barrel contained control solution and the left-hand barrel contained 10 mM glutamate solution throughout, while the concentration in the right-hand barrel was varied. By alternately jumping into the streams from the left and right barrels, any effect of rundown was negated through normalization of the test response to the 10 mM glutamate response. Potency of inhibition by perampanel was assessed by comparing initial peak responses produced by 500 ms fast jumps into 10 mM glutamate in the absence or continuous presence of different concentrations of perampanel. Previous studies have suggested that efficacious doses of perampanel likely result in CSF concentrations that produce only partial AMPAR block ^2^. The concentrations of perampanel we used to assess inhibition of WT GluA2(Q)/γ2 and the GluA2(Q) A643V/γ2 ranged from 30 nM–10 μM. A dose of 0.2 mg/kg in patients (>12 yrs of age) is thought to correspond to a free CSF level of ~250 nM ^3, 4^, close to the *IC*_50_ for hippocampal field EPSPs ^5^. However, given the likely enzyme induction by phenobarbital, it is difficult to estimate free CSF concentrations from the dose administered to our patient (up to 12 mg/day).

**Data analysis.** Records were analyzed using Igor Pro 6.35 (Wavemetrics) with Neuromatic 2.8 ^6^. Entry into desensitization (500 ms application of 10 mM glutamate) and current deactivation (1-2 ms application of 10 mM glutamate) were fitted with the sum of two exponentials and the weighted time constants (τ_w, des_ and τ_w, deact_) calculated, according to:

$$\tau_{w}=\tau_{f}\left( \frac{A_{f}}{A_{f}+ A_{s}} \right)+\tau_{s}\left( \frac{A_{s}}{A_{f}+ A_{s}} \right)$$

where $A_{f}$ and $\tau_{f}$ are the amplitude and time constant of the fast component and $A_{s}$ and $\tau_{s}$ are the amplitude and time constant of the slow component. Recovery from desensitization was assessed by applying desensitizing applications of glutamate (10 mM, 200 ms, −60 mV) followed by single 10 ms test applications at increasing intervals (3–400 ms). For each patch tested, exponential fits of peak current recovery timecourse were used to determine individual τ_recov_ values.

To determine channel conductance non-stationary fluctuation analysis was performed on the decaying phase of currents evoked by 500 ms applications of 10 mM glutamate, as previously described ^7^. The variance for each successive pair of current responses was calculated and the single-channel current (*i*) and total number of channels (*N*) were then determined by plotting the ensemble variance (σ^2^) against mean current (*Ī*) and fitting with a parabolic function:

$$\sigma^{2}=i\bar{I}-\frac{\bar{I}^{2}}{N}+\sigma_{\text{Β}}^{2}$$

where σ_Β_^2^ is the background variance. The weighted mean single-channel conductance was calculated from the single-channel current and the holding potential. Peak open probability (*P*_o, peak_) was calculated by dividing the average peak current by *iN.*

*EC*_50_ and *IC*_50_ values were derived by fitting the Hill equation:

$$I=\frac{I_{\text{max}}}{1+\left( \frac{{EC}_{50}}{[\text{drug}]} \right)^{n_{\text{H}}}}$$

where *I*_max_ is the peak of the fit, *EC*_50_ is the concentration producing the half-maximal response, *n*_H_ is the Hill coefficient and [drug] refers to the concentration of either glutamate or perampanel. Before pooling the data, the *I*_max_ value derived from the fit for each patch was used to rescale the individual datasets. The rescaled values were then averaged and refit for display purposes only.

**Data presentation and statistical analysis.** Statistical analysis was performed using R (version 4.1.1, the R Foundation for Statistical Computing, https://www.r-project.org/). Summary data are presented in **Table 1** as mean ± standard deviation (s.d.) from n patches, together with unpaired mean differences and their 95% confidence intervals. Bias corrected and accelerated confidence intervals were calculated from 5000 bootstrap resamples using the dabestr package in R ^8^. Normality was not tested statistically but gauged from density histograms and/or quantile-quantile plots. All p-values were calculated using a non-parametric two-sided approximate permutation t-test, with 10000 bootstrap replicates using the Coin package in R ^9^. No statistical test was used to predetermine sample sizes; these were based on standards of the field.

**REFERENCES**

1. Coombs I, MacLean D, Jayaraman V, Farrant M, Cull-Candy S. Dual effects of TARP γ-2 on glutamate efficacy can account for AMPA receptor autoinactivation. *Cell Rep*. 2017 20:1123-1135.

2. Rogawski MA, Hanada T. Preclinical pharmacology of perampanel, a selective non-competitive AMPA receptor antagonist. *Acta Neurol Scand Suppl*. 2013:19-24.

3. Gidal BE, Ferry J, Majid O, Hussein Z. Concentration-effect relationships with perampanel in patients with pharmacoresistant partial-onset seizures. *Epilepsia*. 2013 54:1490-1497.

4. Yang Y, Wang G, Chuang A, Hsueh S. Perampanel reduces paroxysmal depolarizing shift and inhibitory synaptic input in excitatory neurons to inhibit epileptic network oscillations. *Br J Pharmacol*. 2020 177:5177-5194.

5. Ceolin L, Bortolotto ZA, Bannister N, Collingridge GL, Lodge D, Volianskis A. A novel anti-epileptic agent, perampanel, selectively inhibits AMPA receptor-mediated synaptic transmission in the hippocampus. *Neurochem Int*. 2012 61:517-522.

6. Rothman JS, Silver RA. NeuroMatic: An integrated open-source software toolkit for acquisition, analysis and simulation of electrophysiological data. *Front Neuroinform*. 2018 12:14.

7. Soto D, Coombs I, Kelly L, Farrant M, Cull-Candy S. Stargazin attenuates intracellular polyamine block of calcium-permeable AMPA receptors. *Nat Neurosci*. 2007 10:1260-1267.

8. Ho J, Tumkaya T, Aryal S, Choi H, Claridge-Chang A. Moving beyond P values: data analysis with estimation graphics. *Nat Methods*. 2019 16:565-566.

9. Hothorn T, Hornik K, van de Wiel M, Zeileis A. Implementing a class of permutatuon tests: The coin package. *Journal of Statistical Software*. 2008 28.
